# Supplementary material for: Identification of photoperiod-induced specific miRNAs in the adrenal glands of Sunite sheep (Ovis aries)
Source: Front Vet Sci. 2022 Jul 22;9:888207. doi: 10.3389/fvets.2022.888207 (PMC9354845; doi:10.3389/fvets.2022.888207)
Supplement: Supplementary file 1 [file Data_Sheet_1.ZIP › Supplementary file/miRDeep2 file.zip!/miRDeep2 file/Novel_Structure/SP42A2/Novel_333.pdf]

Provisional ID	: NC_019460.2_8705
Score total	: 1.7
Score for star read(s)	: -1.3
Score for read counts	: 0
Score for mfe	: 1.4
Score for randfold	: 1.6
Score for cons. seed	:
Total read count	: 138
Mature read count	: 138
Loop read count	: 0
Star read count	: 0

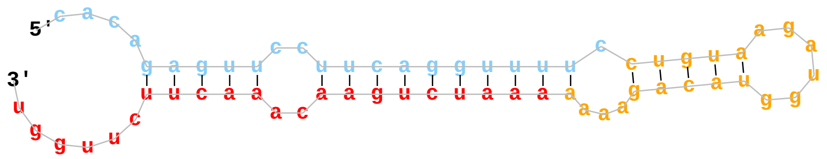

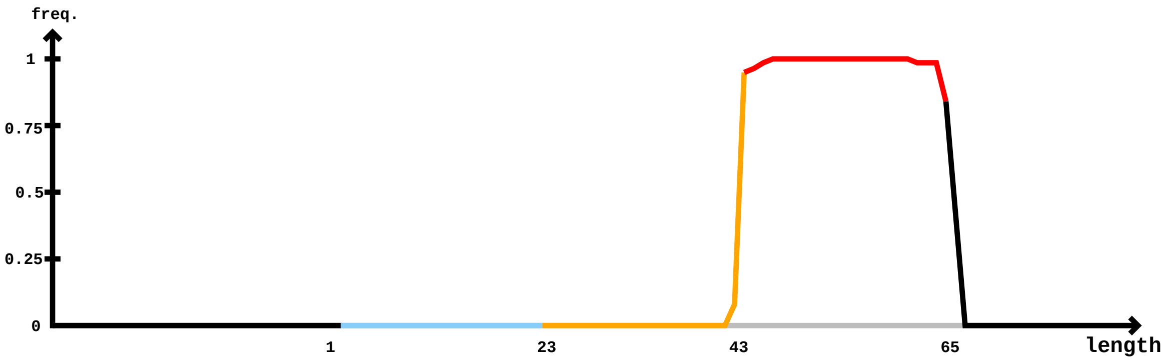

Star Mature

```
5'- gguuaguauuaugaugcacuggguugucсacagaguuccuucagguuuuccuguaagaugguacagaaaaaaaucugaacaaacuucuuggucaacccaauauuaucuguu   -3'    exp
    ..........((((((...(((((((.(((..(((((..((((((((((.(((((......)))))...))))))))))..)))))..))).))))))).)))))).....    reads     mm     sample
    .....................................................................aaaaucugaacaaacuuUu.......................    2         1      seq
    .....................................................................aaaaucugaacaaacuuUuuggu...................    9         1      seq
    ......................................................................aaaucugaacaaacuucuugg....................    1         0      seq
    ......................................................................aaaucugaacaaacuuUuugg....................    19        1      seq
    ......................................................................aaaucugaacaaacuucuuggu...................    1         0      seq
    ......................................................................aaaucugaacaaacuuUuuggu...................    99        1      seq
    .......................................................................aaucugaacaaacuuUuuggu...................    2         1      seq
    ........................................................................aucugaacaaacuuUuuggu...................    3         1      seq
    .........................................................................ucugaacaaacuuUuuggu...................    2         1      seq
```
